# Pharmacotherapeutic options for the treatment of hypertension in pregnancy

**DOI:** 10.1080/14656566.2024.2398602

**Published:** 2024-09-03

**Authors:** Frances Conti-Ramsden, Antonio de Marvao, Lucy C. Chappell

**Affiliations:** a Department of Women and Children’s Health, School of Life Course Sciences, King’s College London, London, UK; b British Heart Foundation Centre of Research Excellence, School of Cardiovascular and Metabolic Medicine and Sciences, King’s College London, London, UK; c Medical Research Council Laboratory of Medical Sciences, Imperial College London, London, UK

**Keywords:** Antihypertensives, beta-blockers, calcium channel blockers, vasodilators, ethnicity, hypertension, pregnancy

## Abstract

**Introduction:**

Hypertensive disorders of pregnancy affect approximately one in 10 pregnancies and are associated with increased risk of adverse fetal, neonatal and maternal outcomes. There is strong evidence that effective treatment of hypertension (blood pressure ≥ 140/90 mmHg), and enhanced monitoring throughout pregnancy reduces these risks.

**Areas covered:**

This article provides a contemporaneous review of treatment of hypertension in pregnancy with antihypertensive agents. We completed a systematic search and review of all meta-analyses and systematic reviews of studies comparing antihypertensives for treatment of pregnancy hypertension in the last five years. We provide a clinically focused summary of when to treat hypertension in pregnancy and which antihypertensive agents can be offered. Special scenarios reviewed include treatment-resistant hypertension and pre-pregnancy antihypertensive optimization.

**Expert opinion:**

Several antihypertensives are considered safe and are known to be effective for treatment of hypertension in pregnancy. Given the current uncertainty as to which antihypertensive(s) are superior for treatment of hypertension in pregnancy, women should be counselled and offered a range of antihypertensive options in keeping with evidence on clinical effectiveness, local context and availability of antihypertensive(s), potential side effect profile, and women’s preference. Further research is required to help guide clinical decision making, and move toward personalized treatment.

## 1. Introduction

**Figure 1.**
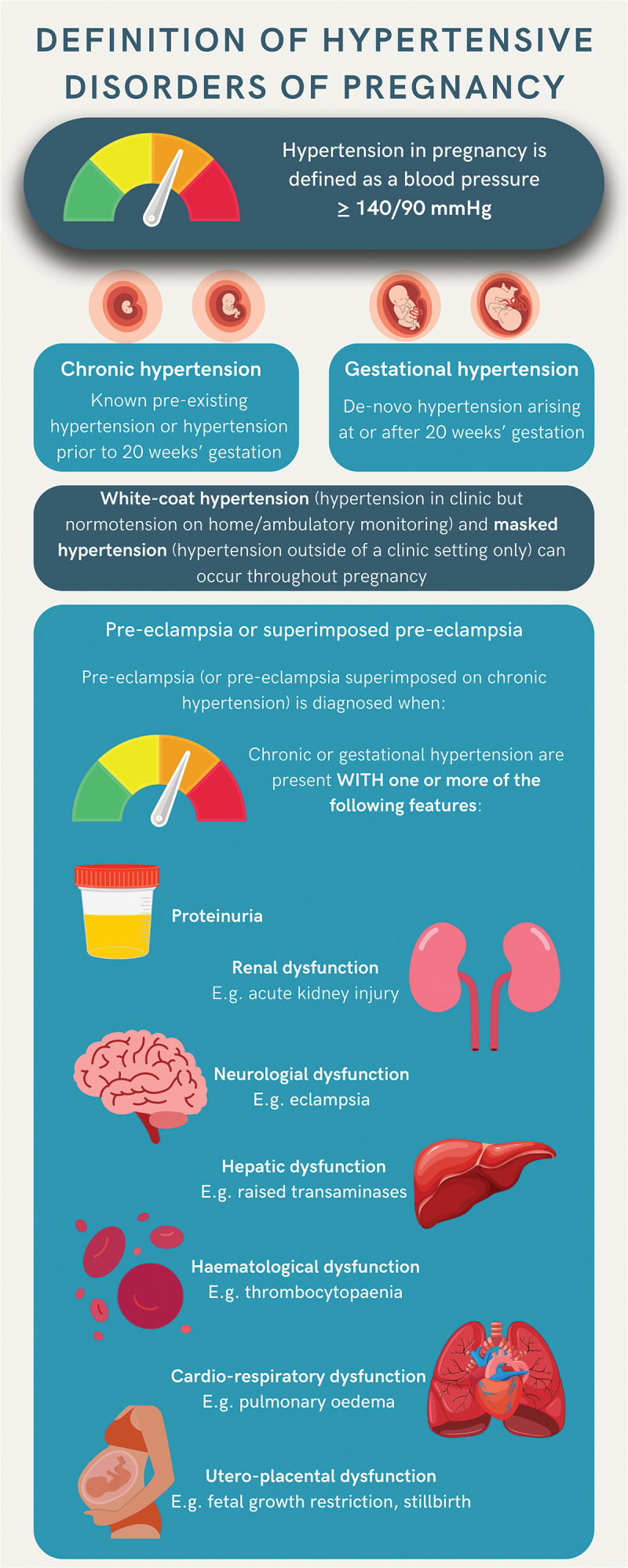
Definition of hypertensive disorders of pregnancy.

Hypertensive disorders of pregnancy (HDP) are the most common complication of pregnancy, estimated to affect 1 in 10 pregnant women. They constitute a spectrum of conditions including hypertensive disorders pre-dating pregnancy and new onset hypertension arising after 20 weeks’ gestation (Figure 1). Pre-existing chronic hypertension can be categorized as essential (without known cause) or secondary to a medical condition. De novo pregnancy hypertension can be isolated (gestational hypertension) or complicated by maternal organ involvement and/or utero-placental dysfunction (pre-eclampsia) (Figure 1). Pre-eclampsia can also arise in the context of preexisting hypertension, known as superimposed pre-eclampsia (Figure 1) [1].

The diagnosis of hypertension in pregnancy requires two readings of a systolic blood pressure (BP) >140 mm Hg and/or a diastolic BP >90 mm Hg, taken a minimum of 4 h apart. Where hypertension is severe (systolic BP 160 mm Hg and/or diastolic BP 110 mm Hg) then two readings 15 minutes apart are sufficient for diagnosis. BP should be measured using a device validated for use in pregnancy and pre-eclampsia. Use of out-of-office measures such as home BP monitoring by women may offer advantages, including confirmation of diagnosis and monitoring of BP on treatment. Home or ambulatory BP monitoring may also uncover white-coat or masked hypertension. White-coat hypertension is defined as hypertension in a clinic setting but normotension (BP < 135/85 mmHg) on home or ambulatory BP monitoring. Masked hypertension is defined as normotension in a clinic setting but hypertension outside of a clinic setting. Both can occur pre-pregnancy and throughout pregnancy (Figure 1).

The association between HDP and adverse maternal and perinatal outcomes is well described, with maternal risks of organ dysfunction, stroke, eclampsia and death, and perinatal risks of placental abruption, stillbirth, growth restriction, preterm birth and admission to a neonatal unit [2,3]. A growing emphasis on BP control and management of severe hypertension in clinical guidelines has aligned with reduction in maternal deaths related to pregnancy hypertension in the UK [4]. While in high-income countries deaths from HDP are now infrequent [4] on a worldwide basis, HDP continue to be a major contributor to maternal and perinatal mortality [5,6].

HDP prevalence varies by region globally and across maternal ethnic groups [7,8], with the highest prevalence in Africa, followed by South-East Asia and the Middle East [9]. Research efforts to understand differences in prevalence of HDP, particularly pre-eclampsia, across geographical areas and ethnic groups, are ongoing [10,11].

### 1.1. Scope of review

This article reviews the treatment of hypertension during pregnancy with pharmacotherapeutic agents. It also covers pre-pregnancy management of antihypertensive drugs, which entails similar considerations as during pregnancy. Postpartum management of pregnancy hypertension is not discussed as pharmacotherapeutic options change substantially following delivery of the infant and transfer of medications into breastmilk must be considered. Non-pharmacological management of hypertension in pregnancy (e.g. exercise and dietary modification) are out of scope and are discussed elsewhere [1,12,13].

Pharmacological treatment of hypertension in pregnancy warrants special consideration for numerous reasons. As HDP are one of the most common complications in pregnancy, antihypertensive agents are some of the most prescribed drug classes in pregnancy. Extrapolation of clinical evidence from the general adult hypertension literature is neither possible nor appropriate. The majority of first-line agents recommended for the treatment of adult hypertension (angiotensin converting enzyme inhibitors, angiotensin receptor blockers and diuretics) are not recommended in pregnancy due to potential teratogenicity and other fetal safety concerns [14–16]. Furthermore, the specific antihypertensive agents commonly used in pregnancy (nifedipine and labetalol) are infrequently used outside of pregnancy, such that trials including these medications to inform drug selection are lacking. Finally, pregnancy entails many physiological changes which strongly influence drug pharmacokinetics, pharmacodynamics and drug tolerability [17].

Pregnancy-specific studies and trials are therefore required to ascertain optimal selection and dosing of antihypertensive agents during pregnancy. However, there is a paucity of evidence to inform optimal antihypertensive choice and dosing in pregnancy [18,19]. This article seeks to provide practical guidance for clinical practice while highlighting the uncertainties in the evidence-base and making recommendations for research. We also note that the management of hypertension in pregnancy is complex, involving obstetric decision-making regarding timing of delivery, location of care and monitoring of the fetus in addition to the use of BP lowering medications. However, these aspects of management are outside of the scope of this article. We point interested readers to a recent review of the management of pre-eclampsia [3], and relevant clinical practice guidelines (e.g. UK NICE NG133 Hypertension in Pregnancy guidelines [12]).

### 1.2. Methodology of review

We have used a systematic approach to this review, searching for and reviewing all meta-analyses, systematic reviews, individual patient data (IPD) analyses, and Cochrane reviews of studies comparing antihypertensives for treatment of pregnancy hypertension in the last five years (2019–2024 inclusive). We have also reviewed latest versions of American College of Obstetricians & Gynaecologists (ACOG) [20,21], The International Society for the Study of Hypertension in Pregnancy (ISSHP) [1], The National Institute for Health and Care Excellence (NICE) (UK) [12], and Society of Obstetric Medicine of Australia and New Zealand (SOMANZ) [13] pregnancy hypertension guidelines. High-quality, randomized-controlled trials and other clinical practice guidelines are discussed where appropriate. Where there are little or no data available to inform recommendations, or where topics have not been covered in guidelines, recommendations have been made on the basis of expert clinical opinion of the authors.

## 2. When to treat hypertension in pregnancy

Early in the first trimester, BP decreases due to vasodilation, likely mediated by prostacyclin and nitric oxide. This reduction reaches its nadir between weeks 20–24, followed by a gradual increase until delivery, although these changes are smaller than previously thought, around 6–7 mmHg magnitude over gestation [22]. This pattern is observed in both normotensive and hypertensive pregnant women and regular monitoring is required to adequately treat this dynamic process. Optimal use of pharmacotherapeutic agents for the treatment of hypertension in pregnancy starts with understanding of both when treatment is indicated (BP treatment initiation thresholds) and the aims of treatment (BP target on treatment).

The need for treatment of severe hypertension (BP ≥ 160/110 mmHg) in pregnancy has long been widely supported due to the well-established association between severe hypertension in pregnancy, particularly with pre-eclampsia, and acute maternal hemorrhagic stroke and aortic dissection [23–25]. However, for many years, there have been concerns in the obstetric community regarding the potential perinatal implications of over-treatment of hypertension in pregnancy, particularly treatment of non-severe or mild-to-moderate hypertension (BP in the range 140/90 mmHg to 159/109 mmHg), especially in women with chronic hypertension [26]. Concerns center on the hypothesis that lowering BP could reduce utero-placental perfusion, particularly in women with cardiovascular systems adapted to chronic hypertension, thereby compromising fetal growth and wellbeing. As such, several clinical practice guidelines have continued to recommend relatively high initiation thresholds for BP treatment in pregnancy (Table 1), with the rationale that exposure to these higher BPs are relatively short-lived in the context of pregnancy. This contrasts with lower BP treatment initiation thresholds that have been recommended in the management of adult hypertension for many years [27,28].

**Table 1.** Comparison of ISSHP [1], ACOG [20,21], NICE [12], and SOMANZ [13] guidelines for hypertension in pregnancy: BP thresholds and targets, and first line antihypertensive agent recommendations.

	ISSHP^1^ (International)	ACOG (US)	NICEC (UK)	SOMANZ (Australasia)
Year of publication or update	2021	2019/2020	2023	2023
**BP treatment thresholds and targets**				
Threshold (mmHg) for pharmacological treatment	≥140/90	≥160/110 (Lower thresholds for women with comorbidities or impaired renal function)	≥140/90	≥140/90
Target value or range (mmHg) on pharmacological treatment	-/85 (diastolic)	120-160/80-110 mmHg (chronic hypertension)	≤135/85	130-140/80-90
**First-line antihypertensive agent recommendations**				
**Non-severe hypertension**				
*Labetalol (oral)*	✓	✓	✓	✓
*Oxprenolol (oral)*	✘	✘	✘	✓
*Nifedipine modified-release (MR) (oral)*	✓	✓	✓	✘
*Methyldopa (oral)*	✓	✘	✘	✓
**Acute-severe hypertension**				
*Labetalol (oral)*	✓	✘	✓	✘
*Labetalol (intravenous)*	✓	✓	✓	✓
*Nifedipine modified release (MR) (oral)*	✓	✓	✓	✓
*Hydralazine (intravenous)*	✓	✓	✓	✓
*Diazoxide (intravenous)*	✘	✘	✘	✓

We now have high-quality evidence that refutes this concern. The Chronic Hypertension and Pregnancy (CHAP) randomized controlled trial (RCT) [29], published in 2022, demonstrated that in women with chronic hypertension, randomization to a BP treatment initiation threshold of 140/90 mmHg versus 160/105 mmHg led to improved maternal and infant outcomes (a reduced risk of a composite outcome of pre-eclampsia, perinatal death, preterm birth <35 weeks’ gestation and placental abruption) without a significant increase in small for gestational age infants. Notably both trial arms had BP targets of < 140/90 mmHg on treatment once the treatment initiation threshold was reached. This demonstrates the importance of the *combination* of optimal BP treatment initiation thresholds *and* BP targets on treatment. Addition of the CHAP trial data to the most recent meta-analyses of randomized controlled trials of treatment versus no treatment has confirmed the benefit of treating mild-to-moderate hypertension in pregnancy, with antihypertensive treatment associated with better maternal and infant outcomes including reduction in severe hypertension, pre-eclampsia, placental abruption, preterm birth and neonatal mortality without an increase in small-for-gestational age infants [30,31].

The CHAP trial findings built on the evidence generated from the Control of Hypertension in Pregnancy Study (CHIPS) randomized controlled trial [32] published in 2015, which reported a lower incidence of severe hypertension in women with chronic and gestational hypertension randomized to tight BP control (target diastolic BP 85 mmHg) compared to those with less-tight control (target diastolic BP 100 mmHg) with no increase in small for gestational age infants between the trial arms. In a secondary analysis, severe hypertension was shown to be associated with a range of adverse maternal and perinatal outcomes including preterm birth and maternal biochemical liver injury [33]. These two large, high-quality trials provide conclusive evidence that initiation of BP treatment at optimal thresholds (i.e. BP ≥ 140/90 mmHg) together with tighter control of BP in pregnancy through appropriate targets are beneficial to the fetus and woman, consistent with findings of a recent updated meta-analysis of randomized controlled trials [34].

It is now clear that all pregnant women with BP ≥ 140/90 mmHg, regardless of the type of hypertension (including those with preexisting chronic hypertension), should be offered antihypertensive agent treatment to lower their BP and thus optimize maternal and fetal outcomes. A BP treatment initiation threshold of ≥ 140/90 mmHg is in keeping with the latest ISSHP [1] NICE UK [12] and SOMANZ [13] pregnancy hypertension guidelines (Table 1) and recommended by the American Society for Maternal-Fetal Medicine [35]. Updated ACOG guidelines are awaited.

The CHIPS trial specified a single target diastolic BP value (85 mmHg). After publication of CHIPS, no further randomized trials have examined effects of lower targets (e.g. <80 mmHg) and/or inclusion of systolic BP targets. A recent meta-analysis of pregnancy outcomes according to achieved BP control on treatment suggested potential additional advantages to tighter BP control with the risk of severe hypertension lowest in those with achieved systolic BP 120–129 mmHg (RR 0.36, 95% CI 0.23–0.57) and systolic BP 130–139 mmHg (RR 0.57, 95% CI 0.48–0.68) compared to those with achieved systolic BP 140-149 mmHg, with no differences in small-for-gestational age infants [36]. As such, while we acknowledge further research is desirable, we pragmatically recommend a BP target of ≤ 135/85 mmHg on treatment, in line with recommendations in the latest updates of ISSHP [1] SOMANZ [13] and NICE UK [12] guidelines (Table 1).

Notably, the clear benefit of tighter control of BP in pregnancy aligns with the trend toward increasingly tight management of BP in the general adult population. In light of growing recognition of the log-linear association between BP and adverse cardiovascular outcomes [28,37], the most recent American College of Cardiology Guidelines for high BP in adults (2017) [28] have re-classified hypertension thresholds. A BP of 120–129/<80 mmHg is now classed as elevated, BP of 130–139/80-89 mmHg as Stage I hypertension and BP of ≥ 140/90 mmHg as Stage 2 hypertension. Antihypertensive treatment is now recommended to be offered to individuals with Stage 1 hypertension with a 10-year cardiovascular risk of 10% or more as primary prevention. There is also a log-linear association between BP at entry to pregnancy and risk of pre-eclampsia [38]. The CHAP study suggests tight BP control may be one mechanism to reduce the risk of incident pre-eclampsia, particularly in high-risk women. Further research is required to determine whether tighter BP control in pregnancy can reduce adverse maternal and fetal outcomes without compromising fetal wellbeing and impact future maternal cardiovascular risk.

## 3. Which pharmacotherapeutic agents can be used in pregnancy?

**Figure 2.**
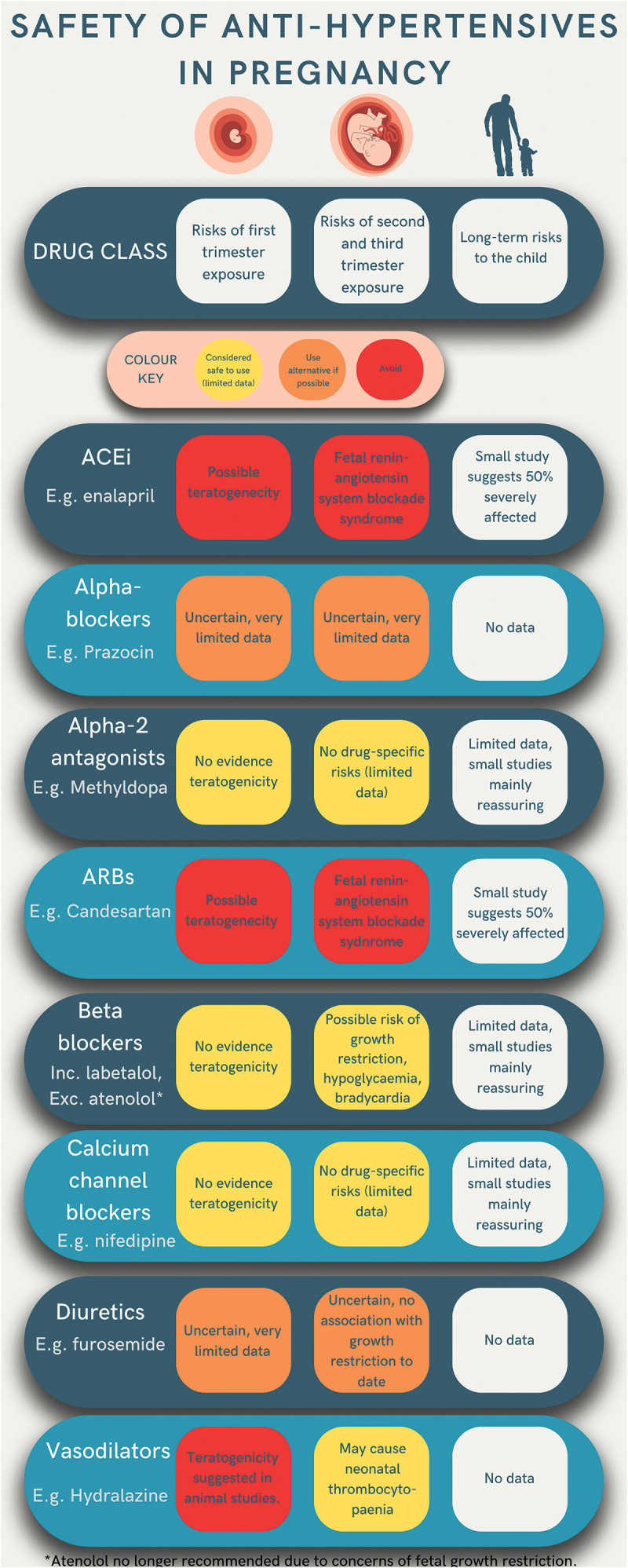
Fetal and infant risks of antihypertensive agent exposure (by drug class).

In pregnancy, the potential maternal and infant benefits of antihypertensive agent therapy discussed above must be weighed against potential adverse effects on the developing fetus and newborn. A summary of confirmed and theoretical risks to the fetus and neonate secondary to exposure to antihypertensive agent drug classes during pregnancy are presented in Figure 2 and Supplementary Table S1.

### 3.1. Calcium channel blockers and beta-blockers

Calcium channel blockers (such as nifedipine), beta-blockers/mixed alpha- and beta-blockers (such as labetalol and oxprenolol), and centrally acting agents (such as methyldopa) have been used for many years in pregnancy and therefore have the most established safety profiles (Figure 2, Supplementary Table S1). While concerns of teratogenicity have previously been raised with all these classes of drugs in older studies, this is likely to reflect higher prevalence of congenital abnormalities in women with chronic hypertension and HDP as opposed to drug-specific effects [39,40], and these drug classes are widely regarded as safe to use in pregnancy [41,42]. However, questions remain unanswered for the drug-specific effects of these classes of drugs on the fetus and neonate which are not answered by published trial data to date [18,43]. In particular, observational data have previously suggested beta-blockers may be associated with growth restriction (particularly atenolol and labetalol) [44] and neonatal beta-blockade causing hypoglycemia and bradycardia [45–47]. It is uncertain as to whether this represents a causal association or whether there is residual confounding with HDP diagnosis in these observational studies, as HDP themselves are strongly associated with growth restriction, reduced birthweight centile and preterm birth [2,3] which are all risk factors for neonatal hypoglycemia.

The 2018 Cochrane review of antihypertensives for treatment of pregnancy did not confirm an association between in-utero beta-blocker exposure as a class effect and growth restriction though confidence intervals do not exclude a detrimental effect on fetal growth [18]. Nor did a more recent network analysis [48]. However, concerns with specific beta-blockers remain, with a network meta-analysis of antihypertensives for chronic hypertension identifying higher risk of small-for-gestational age with atenolol specifically [49]. Concern over neonatal hypoglycemia has been raised, although rates of Neonatal Unit (NNU) admission have not been shown to vary across antihypertensive agent classes [18,48]. These concerns are based on associations between in-utero beta-blocker exposure and infant hypoglycemia demonstrated in large epidemiological studies [45]. Notwithstanding the lack of definitive trial evidence, current clinical practice guidelines in the UK recommend monitoring for infant hypoglycemia in infants exposed to labetalol and other beta-blockers [50]. A recent population-level cohort study of all infant NNU admissions at >34 weeks’ in England and Wales found HDP infants were twice as likely to be admitted for hypoglycemia than non-HDP infants, and one in four HDP infants were admitted for management of hypoglycemia alone [51]. This highlights the need to clarify the infant side-effect profile of commonly used antihypertensive agents in pregnancy. If risk of infant hypoglycemia varies by drug class, infant NNU admissions may be reduced by offering an alternative drug class to women in pregnancy, particularly around the time of birth.

In addition, potential long-term effects of commonly used antihypertensives on child health remain poorly described (Figure 2, Supplementary Table S1). Reassuringly, a recent, large electronic health record linkage study found no association between in-utero exposure to any antihypertensive(s) with developmental outcomes in children at 2.5 years of age with reference to an untreated hypertensive control group (with approximately 70% of children in the exposed group exposed in-utero to a beta-blocker or calcium channel blocker alone) [52], though some further long-term effects are described below.

### 3.2. Angiotensin converting enzyme inhibitors (ACEi) and angiotensin receptor blockers (ARBs)

Strong evidence exists of adverse effects of in-utero fetal exposure to ACEi and ARB, particularly when taken in the second and third trimesters. Use of these medications is particularly discouraged after the first trimester (Figure 2, Supplementary Table S1) and many studies have suggested ACEi and ARBs are teratogens [39,53]. However, more recent studies suggest congenital abnormalities may be driven by underlying HDP as opposed to medication exposure [54,55]. Pre-pregnancy counseling with regards to ACEi and ARB is covered in the pre-pregnancy counseling section of the ‘Special cases of treatment in pregnancy’ heading below. Notably, while ACEi and ARB are largely accepted as contraindicated in pregnancy, studies of prescription data in the US and UK have found as many as 5% of women requiring antihypertensive treatment continue to be prescribed these medications beyond the first trimester [56,57].

### 3.3. Diuretics and alpha-blockers

Diuretics and alpha-blockers have limited safety information in pregnancy due to lack of experience and study of using these medications in pregnancy (Figure 2, Supplementary Table S1). While they are not recommended as first-line options, they may be a suitable choice when medically indicated or in cases of treatment resistant hypertension (see section below ‘Special cases of treatment in pregnancy’). Hypertension in pregnancy guidelines worldwide vary considerably in which medications they recommend avoiding in pregnancy [41]. While all agree ACEi and ARBs should be avoided, some also recommend avoiding diuretics, atenolol (due to concerns of fetal growth restriction) [58], spironolactone and prazosin [41].

## 4. Pharmacotherapeutic options for treatment of mild-to-moderate hypertension in pregnancy

**Figure 3.**
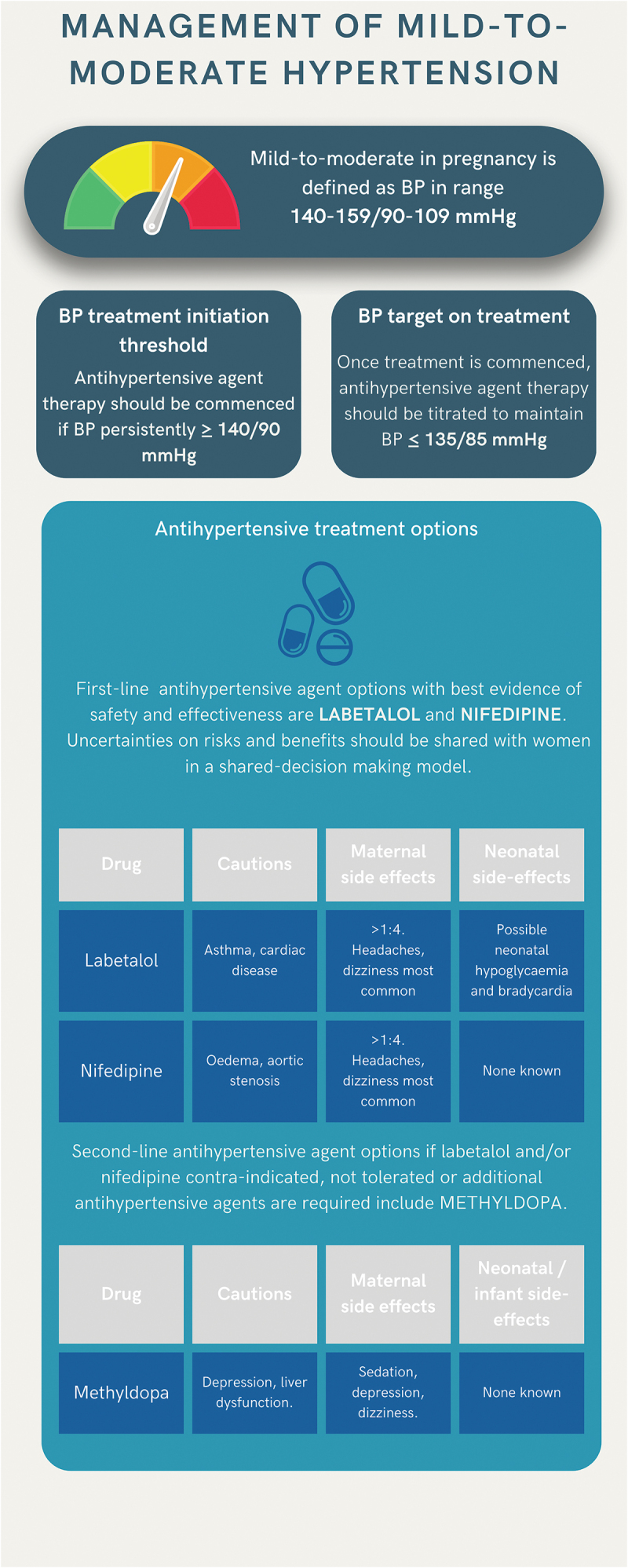
Infographic summarizing management of mild-to-moderate hypertension in pregnancy including definition, BP treatment initiation and treatment thresholds and antihypertensive treatment options.

In this scenario we consider pregnant women presenting with mild-to-moderate hypertension (BP 140–159/80-109 mmHg) including women who have been treated for acute, severe hypertension and are transitioning to oral maintenance therapy (Figure 3). Women should be offered antihypertensive agent therapy promptly after diagnosis, and the aim of treatment is to achieve sustained BP ≤ 135/85 mmHg (BP target). Notably women with mild-to-moderate hypertension in pregnancy may have preexisting (e.g. chronic) hypertension, or de novo hypertension such as gestational hypertension or pre-eclampsia. There is no definitive evidence from randomized controlled trials to suggest antihypertensive agent selection should differ according to underlying hypertensive disorder diagnosis [1], and therefore, the evidence reviewed and recommendations below are applicable to all cases of mild-to-moderate hypertension irrespective of the underlying clinical diagnosis. However, research is ongoing as to whether maternal hemodynamic status or other variables may usefully inform choice of antihypertensive agent (see ‘Expert Opinion’ section) [59].

### 4.1. Evidence to inform initial antihypertensive agent choice in mild-to-moderate hypertension

The latest Cochrane review of antihypertensive drug treatment for mild-to-moderate hypertension in pregnancy, published in 2018, identified 29 trials including 2774 women comparing antihypertensive agents [18] The most commonly used drug classes across studies were beta-blockers (including labetalol, a mixed alpha- and beta-blocker), calcium channel blockers and methyldopa (a centrally acting agent) in keeping with established drug class safety profiles (Figure 2, Supplementary Table S1). The review reported a lower chance of severe hypertension (RR 0.70, 95% confidence interval 0.56 to 0.88, 11 trials, 638 women) in women treated with beta-blockers or calcium channel blockers compared to methyldopa. No differences in risk of pre-eclampsia, neonatal death, small for gestational age infants, or preterm birth was identified when comparing methyldopa, calcium channel blockers and beta-blockers. Notably, confidence intervals were wide and consistent with the possibility of both positive and negative effects, precluding definitive evidence of superiority of an individual agent. Notably, over half of the included trials in the review were old (published before 1990), and many were of poor quality [18]. Only 204 women were included (across two trials) in a head-to-head comparison of the two most commonly recommended agents, labetalol and nifedipine; the review called for large, high-quality, randomized controlled trials to determine which antihypertensive(s) are optimal for treatment of mild-to-moderate hypertension in pregnancy.

A more recent Bayesian and network meta-analysis of randomized trials of antihypertensives for mild-to-moderate hypertension, published in 2022, reported similar results to the Cochrane review in terms of efficacy of beta-blockers, labetalol and calcium channel blockers in preventing severe hypertension [48]. They also reported a lower risk of proteinuria with labetalol compared to calcium channel blockers and methyldopa, although confidence intervals approached 1. Another, less robust meta-analysis published in 2022 has suggested oral nifedipine may have higher efficacy than other agents [60]. A 2020 network meta-analysis specific to chronic hypertension identified nifedipine and methyldopa as having highest efficacy for reducing severe hypertension, and reported atenolol, which has typically not been used for many years due to fetal safety concerns, was associated with fetal growth restriction [49].

An updated search of the World Health Organisation trial registry and ClinicalTrials.gov using terms pregnancy, labetalol and nifedipine on the 15 April 2024 did not identify any further published head-to-head comparisons of antihypertensives for mild-to-moderate hypertension in pregnancy since 2021. One trial (the Giant PANDA study: ISRCTN12792616) investigating nifedipine versus labetalol for hypertension in pregnancy is currently recruiting [61] and should provide robust evidence of which antihypertensive agent is superior from the maternal and infant perspective [62]. One other trial is registered comparing nifedipine versus labetalol for treatment of gestational hypertension (CTRI/2023/11/060095) in a middle-income setting, but is not yet recruiting.

### 4.2. Recommendations for antihypertensive agent choice in mild-to-moderate hypertension

The current lack of definitive evidence to inform optimal first-line antihypertensive agent choice for mild-to-moderate hypertension means there is regional, national and international variation in antihypertensive prescribing and guideline recommendations [41,63]. On the basis of currently available evidence, as beta-blockers and calcium channel blockers are likely to be more effective at avoiding severe hypertension in pregnancy than alternative agents such as methyldopa, with insufficient evidence to recommend one as superior or inferior, we recommend both as potential first-line treatments of mild-to-moderate hypertension in pregnancy in keeping with current ISSHP, ACOG and NICE clinical practice guidelines (Table 1). The specific pharmacotherapeutic agents which have been used for many years in pregnancy with a favorable safety profile to date and are therefore specified in the majority of international clinical practice guidelines as first-line options are oral labetalol and oral modified release nifedipine (Table 1), with suggested dose titration outlined in Table 3 [41]. Formulations, dosing, contraindications, known maternal and infant side effects of labetalol and nifedipine and alternative calcium channel blockers and beta-blockers are presented in Table 2. While labetalol has only one formulation, nifedipine is available in immediate release (IR), modified release (MR) and long acting (LA) formulations. Rapid- or immediate- release forms of nifedipine, which can be sublingual or oral, have been withdrawn in several territories due to risk of severe, unpredictable hypotension [19].

**Table 4.** Dose titration protocol for first-line antihypertensive agents (oral labetalol, oral nifedipine modified release (MR), IV labetalol, IV hydralazine) for treatment of acute, severe hypertension, modeled on 2021 ISSHP guidelines. *T = timepoint in minutes from initiation of treatment.*

		Dose titration if BP target (sustained BP < 160/100 mmHg) not reached					
Drug	Initiation dose (T0)	T 30 minutes	T 60 minutes	T 90 minutes	T 120 minutes	T 150 minutes	T 180 minutes
Labetalol – oral	200 mg	-	200 mg	-	200 mg	-	Consider alternative agent of different drug class
Labetalol – IV intermittent*	10-20 mg	20-40 mg	40-80 mg	40-80 mg	40-80 mg	40-80 mg	
Labetalol – IV infusion	0.5-2 mg/min	Continue infusion	Continue infusion	Continue infusion	Continue infusion	Continue infusion	
Nifedipine MR	10 mg	10 mg	-	10 mg	-	10 mg	
Hydralazine – IV intermittent^†^	5 mg	5-10 mg	5-10 mg	5-10 mg	-	-	

*Maximum dose of IV labetalol is 300 mg total in a treatment course.

^†^Maximum dose of IV hydralazine is 20 mg total in a treatment course.

**Table 2.** Comparison of anti-hypertensives for treatment of hypertension in pregnancy.

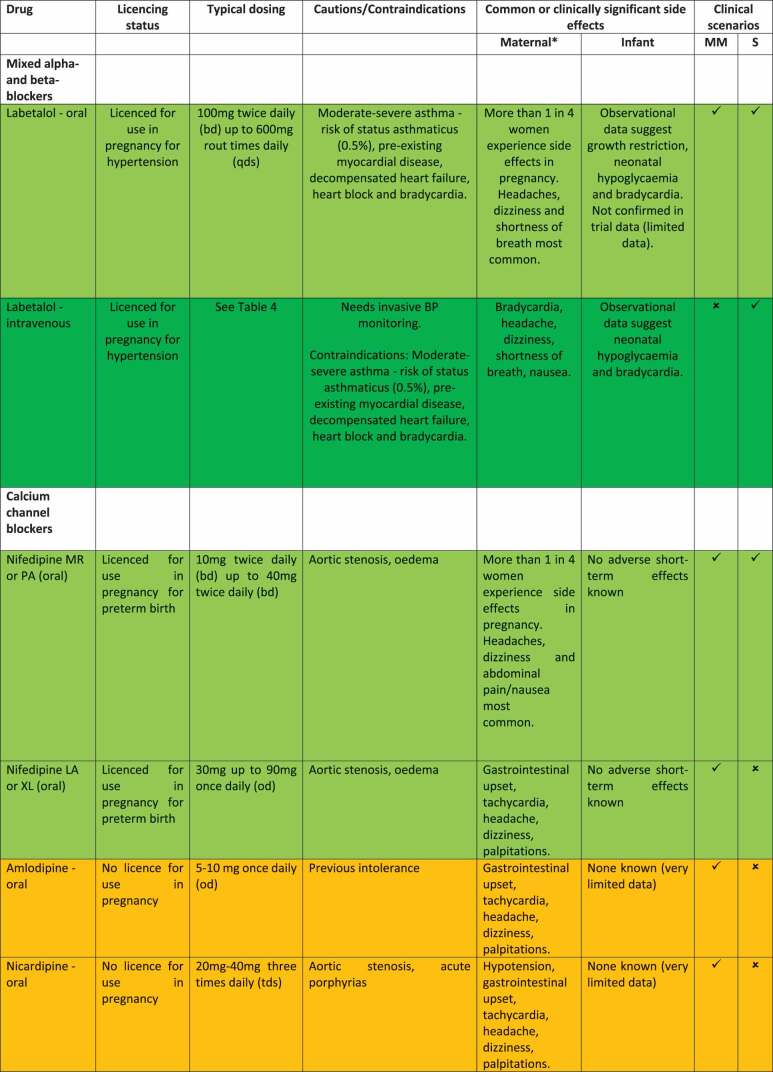
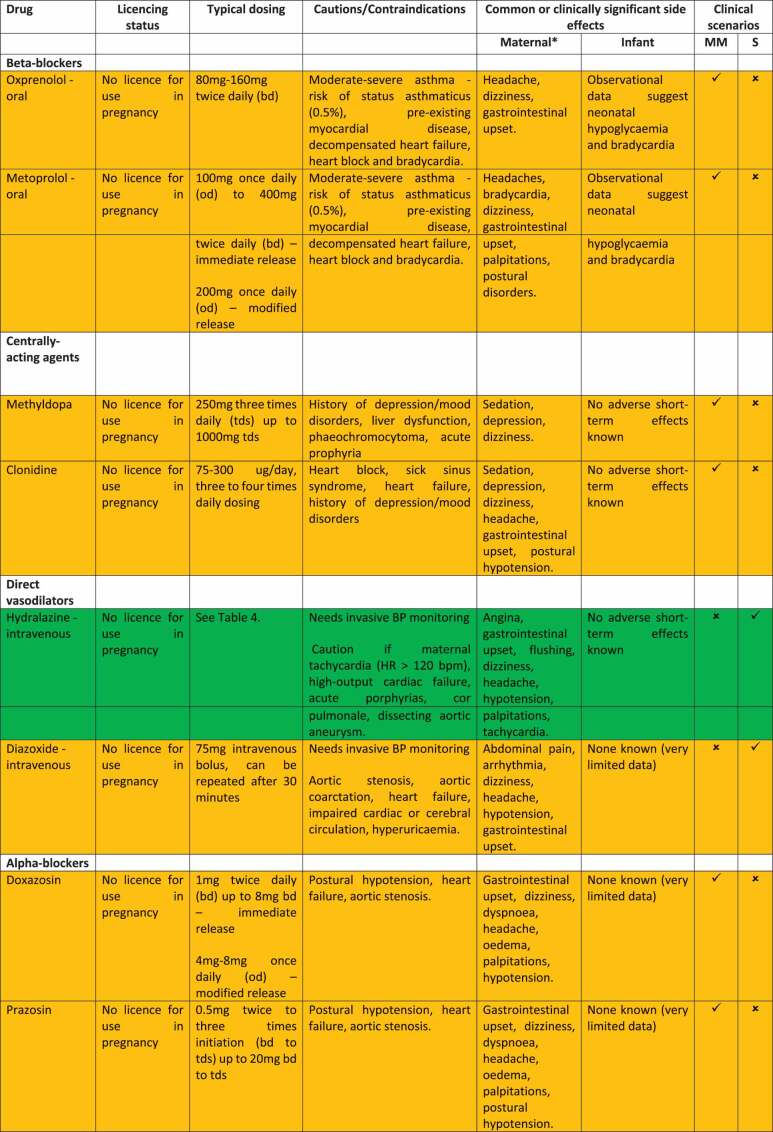

*Side effect profiles in pregnant women specifically are reported for labetalol and nifedipine as per findings of the PANDA feasibility trial [6]. Click or tap here to enter text. For all other drugs, side effect profiles in pregnancy are not available and therefore are reported as per common adverse effects listed by the British National Formulary (https://bnf.nice.org.uk/). Comprehensive lists of possible side-effects of antihypertensives are available in relevant national formularies and manufacturer’s information leaflets. Antihypertensives are colour coded as follows: BRIGHT GREEN = first-line treatment option, DARK GREEN = first-line for acute-severe hypertension only, AMBER = second line treatment option. MM = mild-to-moderate hypertension, S = acute, severe hypertension, MR = modified release, PA = prolonged action, LA = long-acting, XL = extended release.

Oral oxprenolol, amlodipine and nicardipine are other options that have been less studied and may be considered as second-line agents within drug classes depending on antihypertensive agent availability in local contexts (Table 2) [41]. A recent systematic review and meta-analysis of amlodipine versus nifedipine for treatment of hypertension in pregnancy reported amlodipine had comparable efficacy and safety compared to nifedipine and may have fewer side effects [64], though the dosing ceiling (of 10 mg daily) may limit use if hypertension progresses on maximal dosing. Centrally acting alpha-2 antagonists such as methyldopa remain a good second line option, alongside less commonly used drug classes such as alpha-blockers (doxazosin) if beta-blockers and calcium channel blockers are contraindicated, unavailable or not tolerated (Table 2). Further research is required to delineate which antihypertensive(s) are optimal for the woman and baby in mild-to-moderate pregnancy hypertension and is the subject of the ongoing Giant PANDA RCT [62].

### 4.3. Practical clinical consideration 1: shared decision-making

A summary of the management of mild-to-moderate hypertension in pregnancy including BP thresholds for treatment, targets and first-line antihypertensive agent options are illustrated in Figure 3. Choice of first-line antihypertensive agent in each individual case should be guided by a woman’s characteristics, contraindications, and women’s and clinician’s experience and preferences. A shared decision-making model, sharing uncertainties and offering choice to women, may help improve compliance with antihypertensive agents in pregnancy [63]. Shared decision-making infographics for starting antihypertensive agents and antihypertensive agent choices for mild-to-moderate hypertension in pregnancy are available free to download on the UK Action on Pre-eclampsia Charity (APEC) website: https://action-on-pre-eclampsia.org.uk/public-area/high-blood-pressure-in-pregnancy/.

### 4.4. Practical clinical consideration 2: switching antihypertensives

The need for tight BP control, relatively common rate of side effects of antihypertensives in pregnancy estimated as 38% for labetalol and 26% for nifedipine in the PANDA feasibility trial [65], and possibility of suboptimal effectiveness in any individual woman means switching between antihypertensives is a common scenario in pregnancy. Where a drug is not tolerated or is ineffective, women should be offered a first-line drug from a different antihypertensive agent class [1]. For example, if a woman was initially prescribed labetalol, she could be offered nifedipine or vice versa.

There are no data available to guide dosing of antihypertensives when switching between agents in pregnancy. A reasonable approach is to consider percentage equivalent of maximal dose. For example, if a woman is on approximately 50% maximal dose labetalol (e.g. 300 mg labetalol three times daily (TDS)), the clinician could consider switching to 50% maximal dose nifedipine (e.g. 20 mg nifedipine MR twice daily (BD)). However, it is possible that a woman may respond differently to an alternative class of antihypertensive (which may be the reason for switching), so it is also reasonable to be cautious and start the new antihypertensive at a lower percentage maximal dose than the current drug with review at 48 h (often possible virtually if a woman uses home BP monitoring and is otherwise well) and up-titration if required.

### 4.5. Practical clinical consideration 3: down-titrating antihypertensives

Occasionally hypertension spontaneously improves in pregnancy (in a scenario of ‘transient hypertension,’ or where the initial hypertension has been precipitated by a particular situation), and/or a women may not require the initial antihypertensive dose that was started. This can also be seen in scenarios where a woman makes other lifestyle changes (such as reducing salt intake or following a different diet if diagnosed with gestational diabetes) that outside of pregnancy are known to be associated with BP reductions [66]. In the CHIPS trial [32], antihypertensive drugs were down-titrated or stopped if diastolic BP was ≤80 mmHg. The latest version of ISSHP guidelines [1] classify diastolic BP 75-80 mmHg as low normal and <75 mmHg as low. From a practical standpoint, many clinicians down-titrate if low BP are accompanied by symptoms (such as dizziness), and usually down-titrate stepwise.

## 5. Pharmacotherapeutics for treatment of acute, severe hypertension in pregnancy

**Figure 4.**
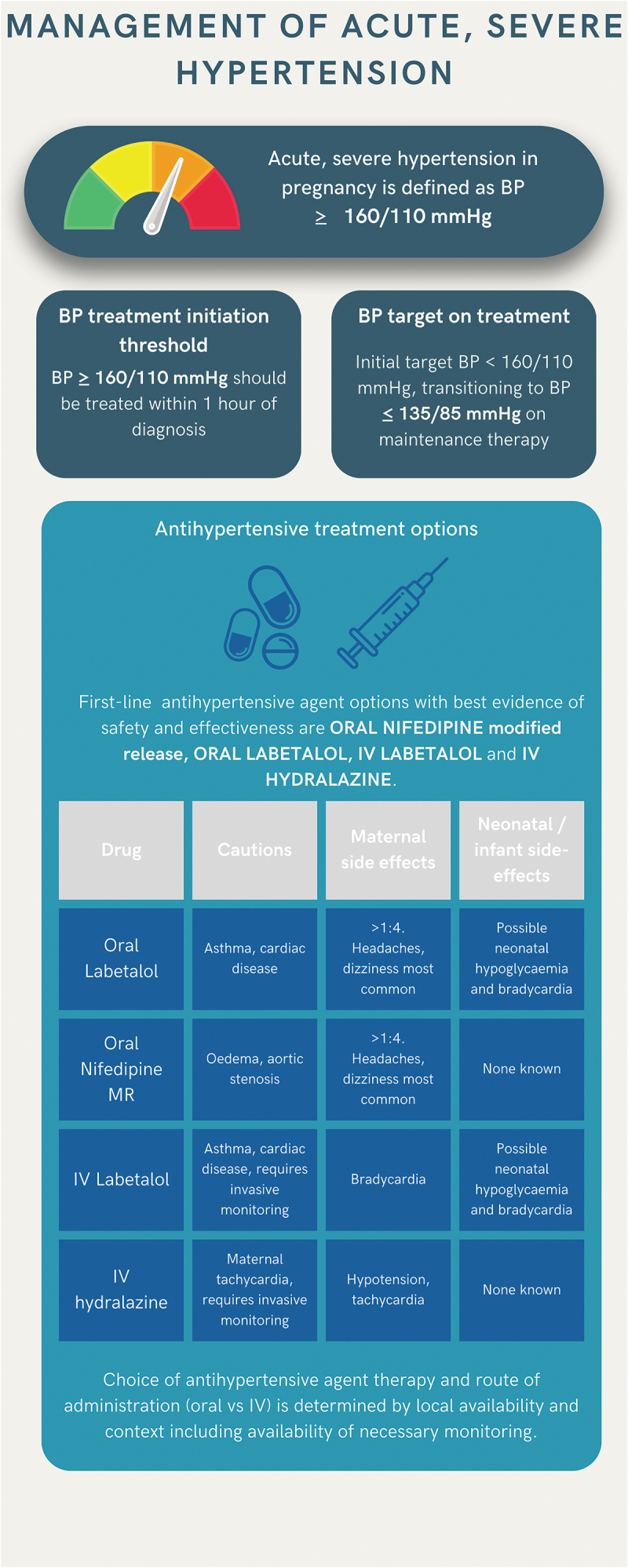
Infographic summarizing management of acute, severe hypertension in pregnancy including definition, BP treatment initiation and treatment thresholds and antihypertensive treatment options.

In this scenario, we consider a pregnant woman presenting with acute, severe hypertension (BP ≥ 160/110 mmHg) in whom stabilization of BP is an urgent concern (Figure 4). Acute, severe hypertension in pregnancy is primarily encountered after 20 weeks’ gestation in the context of pre-eclampsia or gestational hypertension. While severe hypertension may also occur under 20 weeks, in cases of chronic hypertension, presentation is often asymptomatic and the hypertension is likely to have a chronic as opposed to acute time-course, meaning oral antihypertensive agents can be considered in keeping with the recommendations for mild-to-moderate hypertension above. As well as BP control, further investigation for target organ damage and causes of secondary hypertension must be conducted and are discussed in a recent review [67].

### 5.1. BP target timelines in acute, severe hypertension in pregnancy

The risk of maternal stroke is sevenfold higher when severe hypertension occurs with pre-eclampsia as opposed to gestational hypertension, attributed to impaired cerebral autoregulation and endothelial dysfunction in pre-eclampsia, necessitating urgent control of BP [68]. Treatment of acute, severe hypertension in pregnancy should ideally be commenced within one hour of diagnosis to reduce maternal morbidity [69]. The initial aim of treatment is to achieve BP < 160/110 mmHg, and once BP has stabilized to transition to maintenance therapy with a BP target of ≤ 135/85 mmHg. There are few data to guide how quickly BP should be lowered to these targets, particularly in cases of initial systolic BP >200 mmHg [67]. Consensus practice from non-obstetric hypertensive emergencies is to aim to lower systolic BP by no more than 25% in the first hour, aiming for BP < 160/110 mmHg in the next 2–6 hours [67,70]. In pregnancy, theoretical concerns of the adverse effects of rapid reduction of severe hypertension include the potential for fetal bradycardia necessitating delivery and risk of cerebral edema in the context of maternal stroke. A randomized controlled trial of two intravenous (IV) agents (hydralazine and diazoxide) for acute, severe hypertension using a bolus protocol for 1-hour until a target BP of < 140/90 mmHg or 150/100 mmHg was reached, in 124 women, reported only one case of severe maternal hypotension and no other significant maternal adverse effects [71]. Of note, approximately 25% of women in both trial arms had an emergency cesarean section for non-reassuring cardiotocography (CTG) during or after IV antihypertensive administration, and a further 25% had a cesarean section for worsening pre-eclampsia/persistent hypertension [71]. Further research could help elucidate the optimal time frame to reach BP targets in acute, severe hypertension from maternal and infant perspectives [1].

### 5.2. Evidence to inform antihypertensive agent choice in acute, severe hypertension

The most recent Cochrane review of treatment choices for severe hypertension in pregnancy conducted in 2013 was unable to recommend any specific antihypertensive(s) due to inadequate trial data [19] Systematic reviews and meta-analyses comparing antihypertensives for acute, severe hypertension published in 2018, 2019 and 2022 have primarily focused on oral nifedipine, IV hydralazine and IV labetalol [72–74], in keeping with current guidelines (Table 1). All have reported challenges in meta-analyzing the included studies including varying definitions of severe hypertension and differing BP targets across studies [72–74]. The 2018 review and network meta-analysis (32 studies, 3436 women for primary analysis) found no difference in patients achieving target BP for oral nifedipine, IV hydralazine and IV labetalol, though individuals treated with oral nifedipine were observed to reach target BP faster than those receiving IV hydralazine, and use of IV labetalol was associated with fewer instances of maternal tachycardia compared to hydralazine [73]. There was also no difference in stillbirth or neonatal death between the three drugs, though confidence intervals were wide. Furthermore, they observed no clinical benefit in small studies combining drugs including vitamin D and resveratrol with nifedipine [73]. The 2019 review including 17 studies (1591 women) found nifedipine was more effective than hydralazine in treating severe hypertension (OR 4.13, 95% CI 1.01–20.75), with no differences observed in cesarean section rate or maternal side effects [74]. No differences were found in any outcomes comparing oral nifedipine and IV labetalol and IV labetalol and IV hydralazine [74]. The 2022 review, including 17 studies, reported nifedipine had a lower likelihood of persistent hypertension than IV hydralazine (RR 0.40, 95% CI 0.23–0.71) and IV labetalol (RR 0.71, 95% CI 0.52–0.97), with no differences reported in any other maternal or fetal outcomes including cesarean section and stillbirth [72].

The suggestion that oral nifedipine is at least as effective or may be more effective than IV options (hydralazine and labetalol) is also suggested by a 2023 network meta-analysis which included all trials of acute hypertension, which also found oral calcium channel blockers were likely to be the most effective in treating acute, severe hypertension (55 studies, 5518 women), although evidence for superiority was with low certainty and the authors concluded any of oral nifedipine, IV labetalol or IV hydralazine are suitable first-line treatment options [75]. Notably, none of the meta-analyses report the specific formulation of oral nifedipine used (e.g. rapid-release or modified release (MR)/prolonged action (PA)) discriminating only sublingual (presumed rapid-release) versus oral nifedipine in included studies with no subgroup analyses by nifedipine formulation. Rapid-release nifedipine (oral or sublingual) has been withdrawn from use in several countries including the UK due to concerns regarding unpredictable hypotensive effect [76].

The largest randomized controlled trial of antihypertensive treatment for acute, severe hypertension compared oral nifedipine retard (MR), oral labetalol and oral methyldopa in low-resource settings [77]. The trial found all three antihypertensives successfully treated severe hypertension (reducing BP to 120–150/70-100 mmHg) in the majority of women (75% or more), but oral nifedipine MR and oral labetalol were less likely to require administration of a second agent (1% and 3% versus 19% for methyldopa). There were no differences in stillbirths, neonatal deaths or neonatal morbidity between treatment arms. Infants born to women randomized to nifedipine had a higher rate of NNU admission, due to an excess of admissions for low or very low birthweights, though there were no differences in overall birthweights between treatment groups, consistent with a short intervention-to-delivery interval (approximately 24 hours) in all treatment groups [77].

While there have been historical concerns of higher rates of adverse effects in women treated with IV hydralazine, a meta-analysis specifically comparing hydralazine to other agents has also found no evidence of substantial differences in reduction in maternal BP, maternal heart rate or adverse effects compared to nifedipine and labetalol [78].

An updated search of the World Health Organisation trial registry and Clinical trials.gov using the terms pregnancy, hypertension, severe on the 15 April 2024 did not identify any further published head-to-head comparisons of antihypertensives for acute, severe hypertension in pregnancy since 2021, but four trials in middle-income settings investigating oral nifedipine versus IV labetalol are ongoing (CTRI/2023/06/053461, CTRI/2023/01/048873, CTRI/2022/12/048332, NCT06265415). Comparisons between labetalol and hydralazine (NCT06360601) and nitroglycerin (NCT05310929) in middle-income settings are also ongoing.

### 5.3. Recommendations for antihypertensive agent choice in mild-to-moderate hypertension

On the basis of currently available data, there is low-certainty evidence that oral nifedipine MR may be superior to alternative oral agents for acute, severe hypertension. However, uncertainty remains from both perspective of maternal efficacy and infant safety. In keeping with the latest ISSHP and NICE UK guidelines (Table 1), we recommend oral nifedipine MR and oral labetalol (Table 2) as first-line oral therapy options for treatment of acute, severe hypertension and IV hydralazine and IV labetalol as first-line IV therapy options (Table 2). Choice of antihypertensive agent and route is likely to be determined by local context, drug availability and availability of suitable monitoring (Figure 4). Second-line options with more limited evidence of safety include other calcium-channel blockers, other beta-blockers and diazoxide (Table 2). Available data also demonstrate that oral antihypertensive therapy is a reasonable option in acute, severe hypertension as discussed above, and is likely to be more practical where transfer to a setting with appropriate monitoring could cause a delay in receiving treatment and in low-resource settings where IV therapy and concomitant monitoring facilities may not be available [1]. Clinicians can be reassured that initiating treatment for acute, severe hypertension with oral treatment options in any setting is supported by the available evidence.

### 5.4. Practical clinical consideration 1: dose escalation in acute, severe hypertension

A suggested dose titration protocol for acute, severe hypertension, modeled from 2021 ISSHP guidelines [1] is outlined in Table 4.

## 6. Special cases in treatment of hypertension in pregnancy

### 6.1. Treatment resistant hypertension in pregnancy

While the majority of women will achieve BP control on monotherapy, it is not unusual for women with hypertension in pregnancy to require two or more antihypertensive agents to achieve adequate BP control, with an electronic health record study reporting that 30% of women required two or more agents [52]. When a woman does not achieve BP target on the original prescribed antihypertensive drug, the options are i) to increase the dose of the current medication (if not at maximum dose) ii) switch to another agent or iii) add a second agent. There are few published data to guide clinical practice in this scenario. In non-pregnant adult hypertension guidelines [28], there are data supporting using lower doses of two antihypertensives versus a higher dose of one agent to achieve BP control. However, in pregnancy, given uncertainty on true fetal and infant risks of commonly-used antihypertensives, a strategy minimizing the number of antihypertensives a woman is taking is likely to be a prudent approach to reduce potential infant risks (i.e. avoiding low doses of multiple antihypertensives) in the absence of clearer evidence. Therefore, it is reasonable to continue to up-titrate monotherapy up to the at least mid- or high-range dosing (assuming there is evidence of some BP response), prior to adding a second agent. The 2021 ISSHP guidelines recommend consideration of adding a second agent from a different drug class once at least mid- or high-range dosing of the original monotherapy antihypertensive is reached [1]. An example dose titration protocol of labetalol, nifedipine and methyldopa and when to consider transition to dual therapy is summarized in Table 3. If little response is being observed to monotherapy, switching to an alternative agent of another class is also a reasonable approach.

**Table 3.** Dose titration protocol for first-line (oral labetalol and oral nifedipine modified release (MR)) and second line (oral methyldopa) antihypertensive agents for treatment of mild-to-moderate hypertension.

		Dose titration if BP target (sustained BP < 135/85 mmHg) not reached		
Drug	Initiation dose (low)	Dose increase 1	Dose increase 2 (consider adding a second agent as an alternative)	Maximum dose* (recommend adding a second agent as an alternative)
Labetalol – oral	100 mg two to three times daily	200 mg three to four times daily	300 mg three to four times daily	600 mg three to four times daily
Nifedipine MR – oral	10 mg twice daily	20 mg twice daily	30 mg twice daily	40 mg twice daily^†^
Methyldopa – oral	250 mg three times daily	500 mg three times daily	750 mg three times daily	1000 mg three times daily

*Maximum daily dose for indication of adult hypertension as defined in the British National Formulary (BNF).

^†^Maximum daily dose varies internationally between 80-120 mg/day. Clinicians are advised to refer to their national formulary or similar for guidance.

Some women may require three agents, ideally from different drug classes, to control BP, with guidelines typically recommending consideration of delivery of the infant(s) if women are on maximal dose of three antihypertensives [1,12]. Where women require three or more agents early in gestation, clinical judgment on benefits and risks is required, together with consideration of other underlying causes. When three agents are required, additional medications which are commonly used include doxazosin, other beta-blockers and calcium channel blockers (Table 2).

Adherence to medication must also be considered in cases of treatment-resistant hypertension. There is evidence that shared-decision-making can improve adherence to antihypertensives in pregnancy [79]. Adherence to antihypertensives has been shown to be suboptimal in over 90% of pregnant women, with confusion about medication and understanding of risks being primary contributors to non-adherence [80]. Beyond exploring barriers to adherence with women, inpatient admission can be considered for directly observed therapy to determine response to antihypertensive agents [81,82].

### 6.2. Secondary hypertension and medical comorbidities

Treating hypertension in women with secondary hypertension or other concomitant medical conditions presents unique challenges. There is little evidence on the efficacy and safety of antihypertensives used for several secondary hypertension indications and in the context of medical comorbidities. Therefore, a multidisciplinary team with expertise in obstetric medicine and hypertension is necessary to provide a tailored approach.

Asthma is present in around 10% of the population and is a frequent comorbidity in the HDP population. Beta-blockers, such as labetalol, should usually be avoided in patients with asthma, bronchospasm or obstructive airways disease unless no alternative treatment is available. In such cases, the risk of inducing bronchospasm should be appreciated, and appropriate counseling, monitoring and treatment precautions put in place.

Diabetes (preexisting or gestational) is a frequent diagnosis in women with HDP, with insulin sometimes required to achieve tight glycemic control. Beta-blockers may theoretically blunt the early adrenergic symptoms of impending hypoglycemia, but this is very rarely reported in clinical practice [83]. Some caution should be applied in prescribing them in women at high risk of hypoglycemia. Neonates born to diabetic mothers are also well-known to have an increased risk of neonatal hypoglycemia. Late-pregnancy beta-blockers exposure, including labetalol, may cause neonatal hypoglycemia and bradycardia (see Section ‘Which pharmacotherapeutic agents can be used in pregnancy’ above), which could add a ‘second hit’ for neonatal hypoglycemia. Some clinicians are cautious in prescribing beta-blockers in women with co-existing diabetes, if there are particular concerns around maternal or infant hypoglycemia. However, further definitive evidence is required before they are routinely excluded as an antihypertensive choice.

In chronic kidney disease (CKD), the target BP during pregnancy should be 135/85 mmHg or less and this should be achieved with standard pregnancy-compatible antihypertensives.

Primary hyperaldosteronism is the most common cause of secondary hypertension, accounting for at least 5–10% of cases of hypertension in the general population. Limited data are available on the prevalence and on specific management of primary hyperaldosteronism during pregnancy. Mineralocorticoid receptor antagonists (MRA) are preferred for primary hyperaldosteronism treatment outside of pregnancy. Spironolactone is not routinely recommended during pregnancy due to its potential anti-androgenic effect on fetal sexual development. Eplerenone shows lower affinity for androgen receptors compared with spironolactone and a teratogenic effect has not been shown in the few published cases of use [84]. Blockers of the sodium epithelial channel (ENaC), like amiloride, are often used outside pregnancy and no adverse effects have been described in a few case reports in pregnancy [85,86]. On balance, hypertension in pregnancy secondary to primary hyperaldosteronism should be treated with standard pregnancy antihypertensives, with eplerenone or amiloride being considered as second line treatments in cases of uncontrolled hypertension or hypokalaemia despite potassium replacement [87].

Pheochromocytomas are extremely rare in pregnancy, with an estimated prevalence of 1 in 54,000, but are associated with very poor maternal and fetal outcomes if unrecognized. These women should be managed in centers with endocrine, maternal and fetal expertise. Medical management in pregnancy consists of adequate alpha-adrenoceptor blockade with phenoxybenzamine or doxazosin. After adequate alpha-blockade has been achieved for at least a week, beta-blockers or calcium channel blockers can be added to improve BP control. Surgical resection in pregnancy may have to be considered [88].

### 6.3. Pre-pregnancy optimisation of antihypertensives

Women of reproductive age may be taking antihypertensive medication(s) for treatment of chronic hypertension and/or BP control and renoprotection in chronic kidney disease (CKD). Chronic hypertension and CKD are estimated to affect 1–2% and 3% of pregnant women in high-income countries respectively [89–91]. These women should ideally be offered pre-pregnancy counseling with specialist clinicians to optimize medication, control of BP and other disease indicators prior to pregnancy and discuss their risks [12,92].

American, European and UK guidelines for management of adult hypertension (not in pregnancy) all recommend angiotensin converting enzyme inhibitors (ACEi), angiotensin receptor blockers (ARB) and calcium channel blockers (CCB) as first-line treatment options [27,28,93,94]. All three guidelines stipulate ACEi and ARBs are contraindicated in pregnancy, with UK and European guidelines additionally recommending advising against using ACEi and ARBs in women planning a pregnancy [27,93]. In keeping with this, we recommend women taking antihypertensive medications planning a pregnancy or in whom pregnancy is possible (e.g. unreliable contraception) have a discussion about risks and benefits of different antihypertensive agent options. Options include switching to calcium channel blockers which have a favorable safety profile if the woman does become pregnant e.g. nifedipine, or other medications routinely used in pregnancy such as labetalol, which is licensed for use in pregnancy.

In women with CKD, ACEi and ARBs have additional benefits of renoprotection, and given the length of time to falling pregnant is variable as fertility is lower in women with CKD, the risks and benefits of ACEi and ARB to the woman must be weighed against risks of first-trimester embryo exposure. Published guidelines on the management of CKD in pregnancy recommend that while ARBs should be stopped in advance of pregnancy, it is reasonable to consider the option for women to remain on ACEi until an early positive pregnancy test with a clear plan for stopping or conversion to another antihypertensive agent to limit embryonic exposure. This requires specialist assessment and weighing the strength of indication for renin blockade [92], together with a robust plan for early recognition of pregnancy.

## 7. Conclusion

There is strong evidence that hypertension in pregnancy (BP ≥ 140/90 mmHg) should be treated with antihypertensive agents to a BP target of < 135/85 mmHg to optimize maternal and infant outcomes irrespective of underlying diagnosis. While considerable uncertainty on the true fetal and infant risks of commonly used antihypertensive agents in pregnancy remains, calcium channel blockers (particularly nifedipine), beta-blockers/mixed alpha- and beta-blockers (particularly labetalol), centrally-acting agents (particularly methyldopa) and hydralazine, a vasodilator, have the best evidence of safety for use in pregnancy. Other agents which can be considered if required are alpha-blockers such as doxazosin although few data are available to inform clinicians on their in-utero safety profile.

For the treatment of mild-to-moderate or ongoing hypertension in pregnancy (BP 140–159/90-109 mmHg), oral nifedipine or oral labetalol have best evidence of effectiveness and should be offered to women using a shared decision-making approach as first-line treatment options. For the treatment of acute, severe hypertension in pregnancy (BP ≥ 160/110 mmHg), BP should be treated within one hour of diagnosis with oral or IV antihypertensives depending on local context and available monitoring. Oral nifedipine, oral or IV labetalol and IV hydralazine have most evidence of safety and effectiveness and can be offered as first line agents.

Further research is required to determine which antihypertensive(s) are superior for treatment of hypertension in pregnancy from both a maternal and infant perspective, and to offer personalized treatment to individuals in the future.

## 8. Expert opinion

It is clear that further research is required to determine optimal antihypertensive choice in pregnancy. This has been highlighted in the 2020 James Lind Alliance Priority Setting Partnership pregnancy hypertension top 10 research priorities [95] and research recommendations from the 2019 update of the UK NICE Hypertension in Pregnancy Guidelines [12] both of which highlighted the need to define optimal antenatal antihypertensive medication (clinical effectiveness and safety from a maternal and infant perspective) in pregnancy. These questions are being addressed by the Giant PANDA trial, which is randomizing 2300 women to nifedipine versus labetalol and has a co-primary maternal superiority outcome (proportion of BP readings with severe hypertension), and co-primary non-inferiority neonatal outcome (fetal loss, neonatal death or neonatal unit admission). The trial, due to be completed in 2025, will provide high-quality evidence that will inform national and international clinical practice guidelines. However, the challenge of answering this research question was highlighted in the 2022 network meta-analysis of antihypertensives for treatment of mild-to-moderate hypertension. Sample size calculations comparing antihypertensive agents powered on reduction in maternal severe hypertension suggested 2500–10,000 participants/group are necessary to detect a 20% reduction, with prohibitive sample sizes for neonatal outcomes [48]. Therefore further meta-analyses and use of observational and electronic health record data may be required to further delineate antihypertensive maternal and fetal/neonatal risk-benefit profiles.

The latest version of the ISSHP guidelines (2021) [1] included one research recommendation pertaining to antihypertensives: whether hemodynamic-guided antihypertensive therapy can achieve maternal BP control and optimize perinatal outcomes. This reflects the growing interest in personalization of antihypertensive agent treatment in pregnancy, a compelling concept given the relatively short time frame of pregnancy and potential for good BP control to improve maternal and infant outcomes including reduction in pre-eclampsia [29]. Practising obstetricians will be familiar with the experience that some women respond better to labetalol/beta-blockers better than nifedipine/calcium channel blockers or vice versa. While several theories exist as to what may be driving treatment response in pregnancy and variation in clinician prescribing is observed [63], the evidence base is not yet sufficiently robust to have translated into clinical guidelines.

Several factors have been put forward as potential guides of antihypertensive agent in choice in pregnancy including maternal hemodynamics, maternal ethnicity and a growing interest in pharmacogenomics. Maternal hemodynamic profiles have been best characterized in pre-eclampsia, with early-onset pre-eclampsia demonstrating a vasoconstricted, low cardiac output profile (‘hypodynamic’), and late-onset pre-eclampsia typically characterized by a high cardiac output, lower systemic vascular resistance profile (‘hyperdynamic’) [59,96]. It is biologically plausible that beta-blockers may be more effective in individuals with hyperdynamic profiles, while calcium channel blockers and methyldopa may be more effective in those with hypodynamic profiles, as highlighted in a recent European Society of Hypertension position statement on management of HDP [97]. This has been suggested by two small studies investigating hemodynamic-driven prescribing [98,99]. These studies show promise but require validation, with further small studies ongoing (NCT04755764). It is possible that normalizing maternal hemodynamics as an adjunct goal of antihypertensive agent therapy may improve maternal and fetal outcomes [96]. In addition, studies have demonstrated women with vasoconstricted profiles have the highest rates of fetal growth restriction warranting additional study of how to optimize fetal outcomes in this high-risk group [98,99]. However, robust reproducible measures of maternal hemodynamics remain challenging [59]. Further studies investigating maternal biomarkers such as placental growth factor (PlGF), hemodynamics and response to antihypertensive agents may pave the way for future stratified trial designs.

UK NICE guidelines recommend tailoring of antihypertensive treatment for chronic hypertension in non-pregnant adults on the basis of ethnicity and age (NG136, updated 2019) [27] on the premise that there is a higher prevalence of low-renin hypertension in individuals of African ancestry [100,101] leading to an attenuated response to beta-blockers and angiotensin converting enzyme inhibitors (which work primarily by suppressing the renin-angiotensin system), and better response to calcium channel blockers (which work primarily by vasodilation). While little data exist in pregnancy, small studies suggest similar patterns may be observed in pregnancy. In a study of 117 pregnant women with treated chronic hypertension, women classified as being of Black ethnicity had lower renin and aldosterone concentrations across gestation [102]. Furthermore, in a study of 120 pregnant hypertensive women prescribed labetalol monotherapy, BP control (defined as BP < 140/90 mmHg) was almost 20% lower in women of Black versus White ethnic backgrounds [103]. Maternal ethnicity has also been shown to be an independent predictor of labetalol response alongside baseline heart rate and stroke volume index [104].

However, ethnicity is a complex and controversial concept which may encompass primarily social as opposed to biological features [105,106]. Future studies should aim to disentangle whether maternal ethnicity is acting as a proxy for primarily social or genetic variation in this context, with genetic ancestry being one potential route of exploration to elucidate biological determinants [107,108]. In parallel, research and actions to address equity of access to and quality of care across women of all ethnicities and deprivation indices, particularly for women facing multiple disadvantage, are crucial to tackle social determinants of adverse outcomes [109].

Pharmacogenomics is also gaining interest as a precision medicine approach in pre-eclampsia and HDP [110,111]. Studies to date have primarily focused on variants associated with response to any antihypertensive in pre-eclampsia, finding variants in MMP9, TIMP1 and NAMPT are associated with response [110,111]. Only one study has investigated drug-specific response, suggesting variants in the cytochrome P450 CYP2D6 gene (rs1065852) may be associated with efficacy of labetalol treatment [112]. However, studies to date are small and consensus on definition of treatment response is required to progress research in the field [110].

Alongside a better evidence base to inform and personalize antihypertensive agent selection, BP treatment thresholds and targets may continue to evolve in line with out-of-pregnancy hypertension management [28]. Two small trials are currently investigating whether treatment of Stage 1 hypertension (BP 120–139/80-89 mmHg) in early pregnancy (<20 weeks) improves maternal and fetal outcomes (NCT05955040, NCT05989581).

Finally, further research into the pathophysiology of HDP is required to yield targeted treatments for prevention and management of HDP beyond the current options of control of BP and delivery of the fetus and placenta. Notably, small inhibitory RNA (siRNA) based technology is being investigated as a potential therapeutic agent to silence placental expression of placental soluble fms-like tyrosine kinase 1 (sFLT1), a validated diagnostic and prognostic marker which is over-expressed in pre-eclampsia [113]. Large-scale multi-omic studies are also starting to identify the biological pathways in HDP and may lead to further development of targeted therapies for pre-eclampsia and HDP [114].

### 8.1. Viewpoint: management of hypertension in pregnancy in 2035

We anticipate that in 10 years’ time, optimal BP treatment thresholds and targets will have been defined, accepted into clinical practice and used internationally. We speculate that high-quality trial and observational data will have generated a solid evidence base to inform antihypertensive agent selection and counseling in pregnancy. Furthermore, point-of-care measurements or tests, which could include maternal hemodynamics, pharmacogenomics or blood tests (e.g. PlGF, s-Flt) may be used alongside appropriate monitoring such as 24-hour ambulatory BP monitoring or remote BP monitoring to rapidly select and titrate antihypertensives in pregnancy. Novel, targeted therapeutics may also be emerging to modify the course of hypertensive disease in pregnancy.

## Supplementary Material

EOP_HTN_TX_Review_SuppMaterial_revised_20240819.docx

## Article highlights

- Women with hypertension in pregnancy (BP ≥ 140/90 mmHg) should be offered treatment with antihypertensive agents to a BP target of < 135/85 mmHg to optimize maternal and infant outcomes irrespective of underlying diagnosis.
- Oral calcium channel blockers (e.g. nifedipine) and beta-blockers/mixed alpha- & beta-blockers (e.g. labetalol) are considered to be safe, have most evidence of effectiveness for treatment of mild-to-moderate hypertension (BP 140-159/90-109 mmHg) in pregnancy, and should be offered as first-line treatment options.
- Acute, severe hypertension (BP ≥ 160/110 mmHg) in pregnancy should be treated within one hour of diagnosis with oral or IV antihypertensives depending on local context and available monitoring. Oral nifedipine, oral or IV labetalol and IV hydralazine have most evidence of safety and effectiveness and can be offered as first line agents.
- Further research is required to determine which antihypertensive(s) are superior for treatment of hypertension in pregnancy from both a maternal and infant perspective, and to offer personalized treatment to individuals in the future.
