# Supplementary material for: Pharmacotherapeutic options for the treatment of hypertension in pregnancy
Source: Expert Opin Pharmacother. 2024 Sep 3;25(13):1739–58. doi: 10.1080/14656566.2024.2398602 (PMC11881908; doi:10.1080/14656566.2024.2398602)
Supplement: EOP_HTN_TX_Review_SuppMaterial_revised_20240819.docx [file IEOP_A_2398602_SM9566.docx]

**Supplementary materials**

**Supplementary Tables**

**Supplementary Table 1.** Fetal and infant risks of antihypertensive agent class exposure.

*Boxes are highlighted in following colours: YELLOW = considered safe to use during this time period of pregnancy (limited data), ORANGE = use an alternative if possible during this time period of pregnancy, RED = avoid during this period of pregnancy.*

| **Antihypertensive agent drug class** | **Fetal risks 1^st^ trimester exposure** | **Fetal/neonatal risks 2^nd^/3^rd^ trimester exposure** | **Long term risks to infant** | **References** |
| --- | --- | --- | --- | --- |
| ACE inhibitors (ACEi)  E.g. Enalapril | Concerns of potential teratogenicity. Studies have conflicting results; possible that risk of congenital malformation with first-trimester use is driven by underlying hypertension rather than ACEi. However, risk of fetal renin-angiotensin system blockade syndrome exists with first-trimester exposure. | High risk of fetal renin-angiotensin system blockade syndrome including renal failure, oligohydramnios and pulmonary hypoplasia. | Limited data (N = 26 infants) suggest 50% exposed infants mildly to severely affected. | [1–6] |
| Alpha-blockers  E.g. Doxazosin | Uncertain. Case reports, case series and small trials (primarily studying prazosin) have not identified teratogenicity. | Uncertain. Case reports and case series (primarily studying prazosin) have not identified any specific risks. One small randomised trial (2003) found increased risk of stillbirth in Prazosin group (compared to nifedipine). | No data. | [7–10] |
| Alpha-2 antagonists (centrally acting agents)  E.g. Methyldopa | No evidence of teratogenicity specific to antihypertensive agent with first trimester use of methyldopa. | No evidence of increase in miscarriage, small-for-gestational age or preterm birth in infants exposed to methyldopa compared to other commonly used antihypertensives in 2018 Cochrane meta-analysis. | N = 195 children exposed to methyldopa followed up to age 7 had no significant difference in health or physical or mental ability compared to controls. A cohort study of 202 children suggested an association between in-utero methyldopa exposure and sleep disorders. A case-control study of 22 children exposed to clonidine suggested dose-response association with hyperactivity and sleep disorders. | [11–15] |
| Angiotensin II receptor blockers (ARBs)  E.g. Candesartan | Concerns of potential teratogenicity. Studies have conflicting results, possible that risk of congenital malformation with first-trimester use is driven by underlying hypertension. However, risk of fetal renin-angiotensin system blockade syndrome exists with first-trimester exposure. Risk higher with ARB than with ACEi. | High risk of fetal renin-angiotensin system blockade syndrome including renal failure, oligohydramnios and pulmonary hypoplasia. Risk higher than with ACEi. | Limited data (N = 26 infants) suggest 50% exposed infants mildly to severely affected. | [1–6] |
| Mixed alpha- and beta-blockers / Beta-blockers  E.g. Labetalol | No evidence of teratogenicity specific to antihypertensive agent with first-trimester exposure to beta-blockers. | No evidence of increase in miscarriage, small-for-gestational age or preterm birth for infants exposed to beta-blocker drug class compared to other commonly used antihypertensives in 2018 Cochrane meta-analysis However, network metanalysis found higher risk of growth restriction with atenolol specifically. Observational data also demonstrated risk of growth restriction varies by beta-blocker subtype and identified atenolol and labetalol as highest risk. Large cohort studies have also highlighted potential risk of fetal beta-blockade in third trimester causing neonatal hypoglycaemia and bradycardia. Mendelian randomisation study has suggested increased risk of gestational diabetes and lower birthweight with use of beta-blockers in pregnancy [16] | N = 32 infants exposed to labetalol followed up to age 3-7. No differences between IQ compared to unexposed controls. A cohort study of 202 children suggested an association between in-utero labetalol exposure and ADHD. | [12,14,16-23] |
| Calcium channel blockers  E.g. Nifedipine | No evidence of teratogenicity specific to antihypertensive agent with first-trimester exposure to calcium channel blockers. | No evidence of increase in miscarriage, small-for-gestational age or preterm birth for infants exposed to calcium channel blockers compared to other commonly used antihypertensives in 2018 Cochrane meta-analysis. A cohort study suggested increased risk of neonatal seizures with third trimester exposure to calcium channel blockers, but this was not confirmed in a subsequent large, population-based cohort study. | Follow up of N = 115 children who received nifedipine for tocolysis showed no difference in outcomes to 5 years of follow up. | [12, 21, 24-26, 27] |
| Diuretics  E.g. Furosemide | Uncertain. No evidence of teratogenicity in a registry study of 382 exposed pregnancies (primarily furosemide prescription, n = 332/382). | Uncertain. Theoretical risk of disruption of uteroplacental flow due to inhibition of normal pregnancy associated plasma volume expansion. However, no association between diuretic use with small-for gestational age infants has been shown in small studies to date. | No data. | [28-29] |
| Vasodilators  E.g. Hydralazine | Teratogenicity suggested in animal studies, not confirmed in human studies to date. Manufacturers advise avoid in first and second trimester. | Indicated only in severe hypertension (BP ≥160/110 mmHg). May cause neonatal thrombocytopaenia. | No data. | [30] |

**Supplementary References**

[1] D.-K. Li, C. Yang, S. Andrade, V. Tavares and J.R. Ferber, *Maternal exposure to angiotensin converting enzyme inhibitors in the first trimester and risk of malformations in offspring: a retrospective cohort study*, BMJ 343 (2011), pp. d5931.

[2] O. Diav-Citrin, S. Shechtman, Y. Halberstadt, V. Finkel-Pekarsky, R. Wajnberg, J. Arnon et al., *Pregnancy outcome after in utero exposure to angiotensin converting enzyme inhibitors or angiotensin receptor blockers.*, Reprod Toxicol 31 (2011), pp. 540–5.

[3] C.A. Fitton, M.F.C. Steiner, L. Aucott, J.P. Pell, D.F. Mackay, M. Fleming et al., *In-utero exposure to antihypertensive medication and neonatal and child health outcomes: a systematic review.*, J Hypertens 35 (2017), pp. 2123–2137.

[4] J. Fu, G. Tomlinson and D.S. Feig, *Increased risk of major congenital malformations in early pregnancy use of angiotensin‐converting‐enzyme inhibitors and angiotensin‐receptor‐blockers: a meta‐analysis*, Diabetes Metab Res Rev 37 (2021), .

[5] M. Bullo, S. Tschumi, B.S. Bucher, M.G. Bianchetti and G.D. Simonetti, *Pregnancy Outcome Following Exposure to Angiotensin-Converting Enzyme Inhibitors or Angiotensin Receptor Antagonists*, Hypertension 60 (2012), pp. 444–450.

[6] A. Quan, *Fetopathy associated with exposure to angiotensin converting enzyme inhibitors and angiotensin receptor antagonists*, Early Hum Dev 82 (2006), pp. 23–28.

[7] J. Versmissen, B.C.P. Koch, D.W.E. Roofthooft, W. Ten Bosch-Dijksman, A.H. van den Meiracker, L.M. Hanff et al., *Doxazosin treatment of phaeochromocytoma during pregnancy: placental transfer and disposition in breast milk.*, Br J Clin Pharmacol 82 (2016), pp. 568–9.

[8] N. Zitoun, M.K. Campbell, D. Matsui and F. Garcia-Bournissen, *Prospective evaluation of pregnancy outcomes after gestational exposure to prazosin*, Br J Clin Pharmacol 89 (2023), pp. 3324–3329.

[9] D.R. Hall, H.J. Odendaal, D.W. Steyn and M. Smith, *Nifedipine or prazosin as a second agent to control early severe hypertension in pregnancy: a randomised controlled trial*, BJOG 107 (2000), pp. 759–765.

[10] A.D. Davidson, A. Bhat, F. Chu, J.N. Rice, N.A. Nduom and D.S. Cowley, *A systematic review of the use of prazosin in pregnancy and lactation*, Gen Hosp Psychiatry 71 (2021), pp. 134–136.

[11] M. Hoeltzenbein, E. Beck, A.-K. Fietz, J. Wernicke, S. Zinke, A. Kayser et al., *Pregnancy Outcome After First Trimester Use of Methyldopa*, Hypertension 70 (2017), pp. 201–208.

[12] E. Abalos, L. Duley, S. Dw, C. Gialdini, E. Abalos, L. Duley et al., *Antihypertensive drug therapy for mild to moderate hypertension during pregnancy ( Review ) Antihypertensive drug therapy for mild to moderate hypertension during pregnancy*, (2018), .

[13] J. Cockburn, V.A. Moar, M. Ounsted and C.W. Redman, *Final report of study on hypertension during pregnancy: the effects of specific treatment on the growth and development of the children.*, Lancet 1 (1982), pp. 647–9.

[14] P.C.M. Pasker-de Jong, G.A. Zielhuis, M.M.H.J. van Gelder, A. Pellegrino, F.J.M. Gabreëls and T.K.A.B. Eskes, *Antihypertensive treatment during pregnancy and functional development at primary school age in a historical cohort study.*, BJOG 117 (2010), pp. 1080–6.

[15] H.J. Huisjes, M. Hadders-Algra and B.C. Touwen, *Is clonidine a behavioural teratogen in the human?*, Early Hum Dev 14 (1986), pp. 43–8.

[16] J.E.H. Bergman, L.R. Lutke, R.O.B. Gans, M.-C. Addor, I. Barisic, C. Cavero-Carbonell et al., *Beta-Blocker Use in Pregnancy and Risk of Specific Congenital Anomalies: A European Case-Malformed Control Study.*, Drug Saf 41 (2018), pp. 415–427.

[17] R. Lennestål, P. Otterblad Olausson and B. Källén, *Maternal use of antihypertensive drugs in early pregnancy and delivery outcome, notably the presence of congenital heart defects in the infants.*, Eur J Clin Pharmacol 65 (2009), pp. 615–25.

[18] I. Bellos, V. Pergialiotis, A. Papapanagiotou, D. Loutradis and G. Daskalakis, *Comparative efficacy and safety of oral antihypertensive agents in pregnant women with chronic hypertension: a network metaanalysis*, Am J Obstet Gynecol 223 (2020), pp. 525–537.

[19] L. Duan, A. Ng, W. Chen, H.T. Spencer and M. Lee, *Beta‐blocker subtypes and risk of low birth weight in newborns*, The Journal of Clinical Hypertension 20 (2018), pp. 1603–1609.

[20] B.T. Bateman, E. Patorno, R.J. Desai, E.W. Seely, H. Mogun, A. Maeda et al., *Late Pregnancy β Blocker Exposure and Risks of Neonatal Hypoglycemia and Bradycardia.*, Pediatrics 138 (2016), .

[21] R.L. Davis, D. Eastman, H. McPhillips, M.A. Raebel, S.E. Andrade, D. Smith et al., *Risks of congenital malformations and perinatal events among infants exposed to calcium channel and beta-blockers during pregnancy.*, Pharmacoepidemiol Drug Saf 20 (2011), pp. 138–45.

[22] M. Ardissino, E.A.W. Slob, S. Rajasundaram, R.K. Reddy, B. Woolf, J. Girling et al., *Safety of beta-blocker and calcium channel blocker antihypertensive drugs in pregnancy: a Mendelian randomization study.*, BMC Med 20 (2022), pp. 288.

[23] W.S. Chan, G. Koren, M. Barrera, M. Rezvani, D. Knittel-Keren and I. Nulman, *Neurocognitive development of children following in-utero exposure to labetalol for maternal hypertension: a cohort study using a prospectively collected database.*, Hypertens Pregnancy 29 (2010), pp. 271–83.

[24] H.T. Sørensen, A.E. Czeizel, M. Rockenbauer, F.H. Steffensen and J. Olsen, *The risk of limb deficiencies and other congenital abnormalities in children exposed in utero to calcium channel blockers.*, Acta Obstet Gynecol Scand 80 (2001), pp. 397–401.

[25] S.C. Fisher, A.R. Van Zutphen, M.M. Werler, P.A. Romitti, C. Cunniff, M.L. Browne et al., *Maternal antihypertensive medication use and selected birth defects in the National Birth Defects Prevention Study.*, Birth Defects Res 110 (2018), pp. 1433–1442.

[26] B.T. Bateman, K.F. Huybrechts, A. Maeda, R. Desai, E. Patorno, E.W. Seely et al., *Calcium Channel Blocker Exposure in Late Pregnancy and the Risk of Neonatal Seizures.*, Obstetrics and gynecology 126 (2015), pp. 271–278.

[27] T. van Winden, J. Klumper, C. Kleinrouweler, M. Tichelaar, C. Naaktgeboren, T. Nijman et al., *Effects of tocolysis with nifedipine or atosiban on child outcome: follow‐up of the APOSTEL III trial*, BJOG 127 (2020), pp. 1129–1137.

[28] J.A. Van Der Zande, M. Greutmann, D. Tobler, K.P. Ramlakhan, J.M.J. Cornette, R. Hall et al., *Diuretic use in pregnancy: data from the ESC Registry of Pregnancy and Cardiac disease (ROPAC)*, Eur Heart J 44 (2023), .

[29] D. Churchill, G.D. Beevers, S. Meher and C. Rhodes, *Diuretics for preventing pre-eclampsia*, Cochrane Database of Systematic Reviews 2010 (2007), .

[30] E. Widerlöv, I. Karlman and J. Storsäter, *Hydralazine-induced neonatal thrombocytopenia.*, N Engl J Med 303 (1980), pp. 1235.
